# Possible role of locus coeruleus neuronal loss in age-related memory and attention deficits

**DOI:** 10.3389/fnins.2023.1264253

**Published:** 2023-08-24

**Authors:** Alessandra Gargano, Bolanle Fatimat Olabiyi, Michela Palmisano, Andreas Zimmer, Andras Bilkei-Gorzo

**Affiliations:** Medical Faculty, Institute of Molecular Psychiatry, University of Bonn, Venusberg-Campus, Bonn, Germany

**Keywords:** noradrenergic neurons, aging, DSP-4, working memory, attention, episodic memory, cognitive dysfunction, mouse model

## Abstract

**Introduction:**

Aging is associated with a decline in cognitive abilities, including memory and attention. It is generally accepted that age-related histological changes such as increased neuroinflammatory glial activity and a reduction in the number of specific neuronal populations contribute to cognitive aging. Noradrenergic neurons in the locus coeruleus (LC) undergo an approximately 20 % loss during ageing both in humans and mice, but whether this change contributes to cognitive deficits is not known. To address this issue, we asked whether a similar loss of LC neurons in young animals as observed in aged animals impairs memory and attention, cognitive domains that are both influenced by the noradrenergic system and impaired in aging.

**Methods:**

For that, we treated young healthy mice with DSP-4, a toxin that specifically kills LC noradrenergic neurons. We compared the performance of DSP-4 treated young mice with the performance of aged mice in models of attention and memory. To do this, we first determined the dose of DSP-4, which causes a similar 20 % neuronal loss as is typical in aged animals.

**Results:**

Young mice treated with DSP-4 showed impaired attention in the presence of distractor and memory deficits in the 5-choice serial reaction time test (5-CSRTT). Old, untreated mice showed severe deficits in both the 5-CSRTT and in fear extinction tests.

**Discussion:**

Our data now suggest that a reduction in the number of LC neurons contributes to impaired working memory and greater distractibility in attentional tasks but not to deficits in fear extinction. We hypothesize that the moderate loss of LC noradrenergic neurons during aging contributes to attention deficits and working memory impairments.

## 1. Introduction

The alarming increase of the aging population worldwide brings a significant increase in numerous chronic and degenerative diseases, causing substantial socio-economic burden (Ferrucci et al., 2008). In fact, Europe is expected to have the oldest populations in the world in the twenty-first century, with almost one in four Europeans projected to be aged 65 years or older by 2030 (Ferrucci et al., 2008). Because of the gradual increase in aging, there is a rise in age-linked diseases that were less prevalent in the past. Some of the most prevalent central nervous system (CNS) diseases related to aging consistent of progressive neurodegenerative cognitive impairment ultimately leading to Alzheimer’s disease (AD), with incidence of 10% in humans older than 65 years (Alzheimer's Association Report, 2023).

Even normal, healthy aging is associated with a decline of attention and cognitive performance (Glisky, 2007) leading to increasing deficits in learning and memory. However, there is a large individual variability in the onset and speed of cognitive loss: While many senior citizens still enjoy their cognitive abilities at an advanced age, others may show signs of cognitive impairment and increased risk of neurodegenerative diseases like Alzheimer’s or Parkinsons’s disease early in their life. The onset and progression of age-related decline in brain functions also differs strongly between the cognitive domains in humans (Glisky, 2007) and also in rodents (Bilkei-Gorzo et al., 2012). The reason for this large variance is partly the differences in the ability to recruit additional brain areas for task solving, which can partially counterbalance the effect of functional decline (Eyler et al., 2011). Another factor, which may contribute to the differences in the effect of aging on cognitive functions, is the large variance in the sensitivity to age-related changes between brain areas andneuronal types (Double et al., 2010; Small et al., 2012).

There are a wide range of molecular and histological changes in the aging brain, including increased levels of toxic metabolic end products, misfolded macromolecules and non-functional organelles, increased pro-inflammatory activity of glial cells and depletion of catecholaminergic neurons. Although these changes are known to underlie age-related functional deficits, it is not known whether and at what level these individual factors influence cognitive aging. Identification of the critical factors would be key for targeted therapy of cognitive deficits in aging. One of the most sensitive neuronal populations to aging is the noradrenergic neurons in the locus coeruleus., therefore in the present work we focused on this brain area. Particularly, on the potential consequences of the loss of noradrenergic neurons in the locus coeruleus (LC) on learning and attention, which are known to be impaired in aging and are influenced by the activity of the noradrenergic system.

The LC represents the major source of noradrenaline (NE) in the central nervous system, comprising just 45–50 thousand neurons in healthy young adult humans (Sharma et al., 2010) and roughly 1,500 in adult mice (Sturrock and Rao, 1985). Noradrenergic terminals richly innervate the cortex, hippocampus, amygdala, hypothalamus and brainstem (Westlund and Dan Coulter, 1980; Gaspar et al., 1989; Sadikot and Parent, 1990; Ginsberg et al., 1993; Radley et al., 2008). LC terminals have synaptic (Papadopoulos et al., 1987) and also non-synaptic (Beaudet and Descarries, 1978) release sites, where they bind and activate noradrenergic receptors. There are two subfamilies of noradrenergic receptors: those in the α subfamily are Gq (α1, often associated with excitation) or Gi (α2, often associated with inhibition) coupled, whereas members of the β subfamily are Gs-coupled metabotropic receptors (Szabadi, 2013). Activation of neuronal noradrenergic receptors leads to long-term alterations in synaptic strength and gene transcription, particularly within the hippocampus and prefrontal cortex, influencing memory formation (Sara, 2009) and consolidation (Gibbs and Summers, 2002). Most probably noradrenergic signaling influences memory not only directly but also indirectly, through the regulation of arousal. Lower or higher arousal than optimal leads to impaired learning ability. Reduced arousal is associated with drowsiness and sleepiness, whereas high arousal is associated with distractibility and anxiety. Noradrenergic neurons in the LC are responsible for setting of the optimal arousal level for different life situations and tasks, thus for the optimization of performance (Aston-Jones and Cohen, 2005). It is hypothesized that noradrenergic neurons act as a temporal attention filter enabling focused attention in the presence of stimuli associated with high reward or necessary for avoidance and for the optimal balance between goal-oriented and exploratory behavior (Aston-Jones and Cohen, 2005).

Evidence from several studies suggests that noradrenergic dysfunction is involved in the development of age-related cognitive deficits. Experimental disruption of LC-noradrenergic transmission in animal models generally led to an impaired performance in tasks designed to measure attention-related processes (Sara, 1985) supporting the notion that lesions to the dorsal noradrenergic bundle interfere with the animal’s ability to select stimuli and to maintain attention once selected (Marien et al., 2004). Indeed, noradrenaline-depleted rats performed as well as non-lesion control animals in the visual discrimination task, but when irrelevant stimuli were introduced into the discrimination array, the depleted group was severely impaired (Oke and Adams, 1978). Noradrenaline-depleted animals showed significant impairment in performing the task also when the trials were presented unpredictably and more rapidly, or when a white noise arousal stimulus was presented (Carli et al., 1983) underlying again the importance of noradrenergic signaling in selective attention. A number of animal studies reported that depletion of NA differently affects cognitive domains: it has no or minimal effect in test of spatial memory including Morris water-maze (Hagan, 1983) or radial arm maze tests (Chrobak et al., 1985) whereas in emotionally arousing models like in the inhibitory avoidance test it impaired long term retention (Prado de Carvalho and Zornetzer, 1981) and performance in the fear conditioning test (Giustino and Maren, 2018). Moreover, NA depletion enhanced the effect of the cholinergic antagonist scopolamine (Decker and Gallagher, 1987) whereas an increase of NA signaling improved performance of aged rodents in the inhibitory avoidance (Zornetzer, 1985) test.

Noradrenergic neurons in the LC can protect against brain aging and the progression of neurodegenerative diseases by influencing glial activity. The pro-inflammatory activity of glia cells increases during aging, which contributes to the aging process and has a pathogenic role in neurodegenerative processes. Noradrenalin exerts potent anti-inflammatory effects (Madrigal et al., 2005) through activating microglial and astrocytic adrenoceptors and on microglia (Szabó et al., 1997) and astrocytes (Frohman et al., 1988). In the brain, the anti-inflammatory effect of NA is mediated via β2 adrenergic receptors (Loop et al., 2004) and involves a reduced activity of NFkB (Gavrilyuk et al., 2002; Heneka et al., 2003). The age-related decline in the number of LC noradrenergic neurons can contribute to the enhanced glial activity and to the increasing pro-inflammatory milieu in the aging brain. Indeed, in our previous work we showed that depletion of NA neurons with DSP-4 treatment in APP23 mice enhanced pro-inflammatory activity of glia cells, augmented amyloid deposition and neurodegeneration, increased memory deficits (Heneka et al., 2010). Whether decreased noradrenergic cell number contributes to the neuroinflammatory changes associated with normal aging is not known.

It seems well-known that the disruption of the noradrenergic system is linked to the onset and progression of neurodegenerative diseases. It has been demonstrated that the LC undergoes an approximately 60% neuronal loss in patients suffering from Parkinson’s disease (PD) and Alzheimer’s disease (AD) even from the early stage of the disease (Braak et al., 2003, 2011; Ordureau et al., 2015; Hou et al., 2018; Reddy and Oliver, 2019). However, whether or not LC neurons are also lost during physiological aging is still debated (Flood and Coleman, 1988; Mouton et al., 1994; Manaye et al., 1995). A recent *in vivo* human study showed a correlation between LC signal intensity values and age, revealing an age-related decline in LC signal intensity values for the first time (Liu et al., 2019). For mice the results were controversial (Leslie et al., 1985; Sturrock and Rao, 1985; Tatton et al., 1991; Szot et al., 2009) but in our recent study, using unbiased stereological counting, we found an age-related approximately 20% decline in the number of noradrenergic neurons in the LC of C57BL/6 J mice (Gargano et al., 2021).

In the present study, we wanted to understand whether this limited decrease in the number of LC noradrenergic neurons *per se* affects cognitive abilities. To do this, we used the noradrenergic neuron-specific toxin DSP-4 to reduce the number of noradrenergic neurons in young animals to levels typical of old mice. We first determined the dose of DSP-4 required to induce a loss of LC neurons in young mice similar to that seen in old mice. We then investigated whether this approximately 20% reduction in the number of LC noradrenergic neurons in DSP-4-treated young animals leads to similar deficits in fear memory using contextual fear conditioning (CFC) and in attention using the 5-choice serial reaction time test (5-CSRTT) as observed in aged mice. We focused on these cognitive domains because they are impaired in old individuals and are regulated by the noradrenergic system.

## 2. Materials and methods

### 2.1. Animals

C57BL/6 J wild-type (WT) male mice were used. Animals were kept under a reversed light cycle with food and water *ad libitum* and bred under specific-pathogen-free conditions in the main animal facility of the University Hospital Bonn (UKB). Mice were housed in groups of 4–5 until the behavioral tests during which they were single-caged. Different cohorts of mice were used for the dose-seeking experiment and for the two behavioral studies. In order to establish the dose of DSP-4 toxin to use, we injected intraperitoneally a cohort of 5 weeks-old C57BL/6 J wild-type mice. For the behavioral studies, we tested a cohort of 3-month-old (young) vs. 20-month-old (old) C57BL/6 J wild-type animals and then a separate cohort of 4-month-old animals treated either with the DSP-4 toxin or a vehicle solution. Animals were randomly assigned to treatment groups. The experimenter was blind to the treatment, although the difference between the age groups was obvious. Care of the animals and conduction of the experiments followed the guidelines of the European Communities Directive 86/609/EEC and the German Animal Protection Law regulating animal research. Animal experiments were approved by the Landesamt fuer Natur, Umwelt und Verbraucherschutz Nordrhein-Westfalen (LANUV NRW; 81-02.04.2020.A360).

### 2.2. DSP-4 treatments

DSP-4 (N-(2-Chloroethyl)-N-ethyl-2-bromobenzylamine hydrochloride) is a widely used neurotoxin, able to bind and kill noradrenergic neurons selectively. Because of its instability, DSP-4 was freshly prepared before use by dissolving it in MilliQ water at pH 5. To determine the dose of DSP-4 toxin needed to cause the 20% of reduction in the number of LC noradrenergic neurons, 6 animals per dose were intraperitoneally injected (vehicle, 6.25, 12.5, 25, 50 mg/kg, vehicle). After 60 days the animals were sacrificed and the number of LC TH-positive cells counted. For the behavioral studies, a single intraperitoneal injection of DSP-4 (14 mg/kg) was used (Fritschy and Grzanna, 1991; Ross and Stenfors, 2015).

### 2.3. Behavioral studies

#### 2.3.1. Contextual fear conditioning

For the Contextual Fear Conditioning (CFC) we used the UGO BASILE ANY-maze controlled Fear Conditioning System, consisting of: FC-Unit (55 × 60 × 57 cm), FC-Cage with electrified floor (24 × 20 × 30 cm), an individual controller on-board (version 1.1.6), and the ANY-maze software (ANY-maze 6.3 ©1999-2021 Stoelting Co.).

The total duration of the fear conditioning was 6 min: it started with a 3 min free exploration stage in the FC-Cage (cage light: 30 lux; white noise: 10 units), and a 3 min conditioning stage where 2 s foot shock (intensity: 0.8 mA) was presented at every minute. For the following 4 days—the extinction phase—mice were returned to the same FC-Cage for 3 min in the absence of the unconditioned stimulus. The behavior of mice was continuously recorded by an automatic video system and the duration of freezing reaction was registered by the ANY-maze system (Curzon et al., 2009; Shoji et al., 2014).

#### 2.3.2. 5-choice serial reaction time test

For the 5-Choice Serial Reaction Time Test (5-CSRTT) we used the 5–9 Holes Box Operant Conditioning (LE507-76-0001) from Panlab Harvard Apparatus and the PACKWIN software (Panlab, v2.0.07). The day before the start of the test the mice were food deprived. For the whole duration of the test the animals received the 80% of their regular food intake (FI), based on their daily body weight (BW).

Animals were trained to respond to a brief, unpredictable visual stimulus (hole illumination) presented in one of five locations in order to obtain a food reward (reinforcement). The test consisted of 6 phases of increasing difficulty. Each session lasted 30 min and progressing from one phase to the next required meeting the criteria for at least two consecutive days. A detailed description of the different phases and the criteria used is summarized in Table 1. During the last phase of the test, animals were exposed to a distractor consisting of: a sound (music), a smell (a cotton swab soaked in lemon extract) and a visual element (stroboscopic light). The learning capacity was measured as days needed to pass each phase, while the choice accuracy was measured in terms of correct, incorrect and omitted answers in the presence or absence of the distractor (Ciampoli et al., 2017).

**Table 1:** 5-CSRTT phases.

Phase	ITI^a^ (sec)	SD^b^ (sec)	LH^c^ (sec)	Criteria
Training to Phase 1	/	/	/	>20 reinforcements +20 detections at the food dispenser for 3 days OR >25 reinforcements + >25 detections at the food dispenser for 2 days
Phase 1 to Phase 2	5	20	10	>20 correct answers, 20% of the total trials^*^
Phase 2 to Phase 3	5	10	10	>30 correct answers, 30% of the total trials
Phase 3 to Phase 4	5	8	10	>40 correct answers, 80% accuracy^**^
Phase 4 to Phase 5	5	4	5	>40 correct answers, 80% accuracy
Phase 5	5	2	5	>45 correct answers, 80% accuracy

a ITI = inter-trial interval.

b SD = stimulus duration.

c LH = limited hold time.

* Total trials = no. correct + incorrect + omitted answers.

** Accuracy = no. correct answers/no. correct + incorrect answers.

### 2.4. Tissue preparation

Mice were deeply anesthetized with a mixture of ketamine and xylazine and transcardially perfused with phosphate buffered saline (PBS) followed by 4% formaldehyde. The isolated brains were post-fixed 2 h in 4% formaldehyde solution, kept in 20% sucrose overnight for cryoprotection, snap frozen in dry ice-cooled isopentane and stored at −80°C. 18 μm thick coronal slices were sectioned using a cryostat (CM3050 S, Leica, Wetzler, Germany), mounted on glass slides and kept at −80°C until further use.

### 2.5. Microscopy

Immuno-stainings were carried out as previously described (Gargano et al., 2021). Briefly, frozen sections were permeabilized in PBS containing 0.5% Triton X-100 and blocked in PBS containing 3% bovine serum albumin. Next, slices were incubated overnight at 4°C with the primary antibody: sheep anti-TH (1:1000, Abcam, Cambridge, UK), mouse anti-TNFα (1:100, Abcam, Cambridge, UK) rabbit anti-Iba1 (1:2000, Wako, Osaka, Japan). After washing, slides were incubated for 2 h with the respective secondary antibody: AF488 anti-sheep, AF647 anti-mouse, AF568 anti-rabbit. Both primary and secondary antibodies were diluted in PBS containing 0.5% bovine serum albumin and 0.05% Triton X-100. Finally, slices were mounted with 4′,6-diamidino-2 phenylindole (DAPI, Southern Biotechnology Associates, Birmingham, AL, United States) and covered and stored at 4°C. Images were obtained with an LSM SP8 confocal microscope (Leica, Wetzler, Germany). For the NET staining, TBS instead of PBS was used and the slices were subjected to an antigen retrieval step. Negative controls were stained only with the secondary antibodies. Iba1 density, cell body size (area) together with Iba1 and TNFα signal intensities were analyzed within the Iba1-positive microglia within the LC in both hemispheres with Fiji software (Ver. 2.1.0/1.53c, NIH, Bethesda, MD, United States).

For light microscopy the processing of the tissues was alike, except we used biotinylated donkey anti-sheep secondary antibody (1:500, Abcam, Cambridge, UK). Slides were incubated with ABC-reagent (Vectastain, Vector Laboratories, CA, United States) and afterwards immersed in 0.5 mg/mL DAB and 0.5 mg/mL NH4Ni(SO4)2 in 50 mM Tris pH 7.3. The reaction was started by addition of H2O2 and stopped by 50 mM Tris. Slides were rinsed in MilliQ water and dehydrated with increasing concentrations of ethanol and xylol. The slices were mounted with Roti Histokitt II mounting medium (Carl Roth GmbH, Karlsruhe, Germany), covered and stored at 4°C. Images were obtained with Axio Imager M2 microscope (Zeiss, Oberkochen, Germany) with 20× objective lens.

For the stereological quantification of TH-positive cells, every 4th slice of the region of interest was collected for a total of 8 slices per sample. Then, we stained for TH immunoreactivity. The total number of TH-positive neurons in the LC region was estimated manually using the plugin Cell Counter from Fiji software (Gargano et al., 2021).

### 2.6. Statistics

The number of animals or samples is indicated in the figure legends. All the data are presented as means ± SEM and statistical analysis was done using the Prism software (Ver. 9.4.1, GraphPad Software, San Diego, CA, United States). Data distribution was analyzed using the D’Agostino and Pearson normality test. Significant outliers were identified and excluded by using Grubb’s test. The DSP-4 dose-response relation was calculated using the simple linear regression model. Statistical significance was determined by Student *t*-test, Mann–Whitney test, ordinary one-way/two-way ANOVA or Kruskal-Wallis test. Šidák’s correction and Tukey’s, Dunn’s or Dunnett’s multiple comparisons test were used for *post hoc* analysis.

## 3. Results

### 3.1. Determination of the DSP-4 dose that induces similar reduction in the number of LC noradrenergic neurons as aging

**Figure 1:**
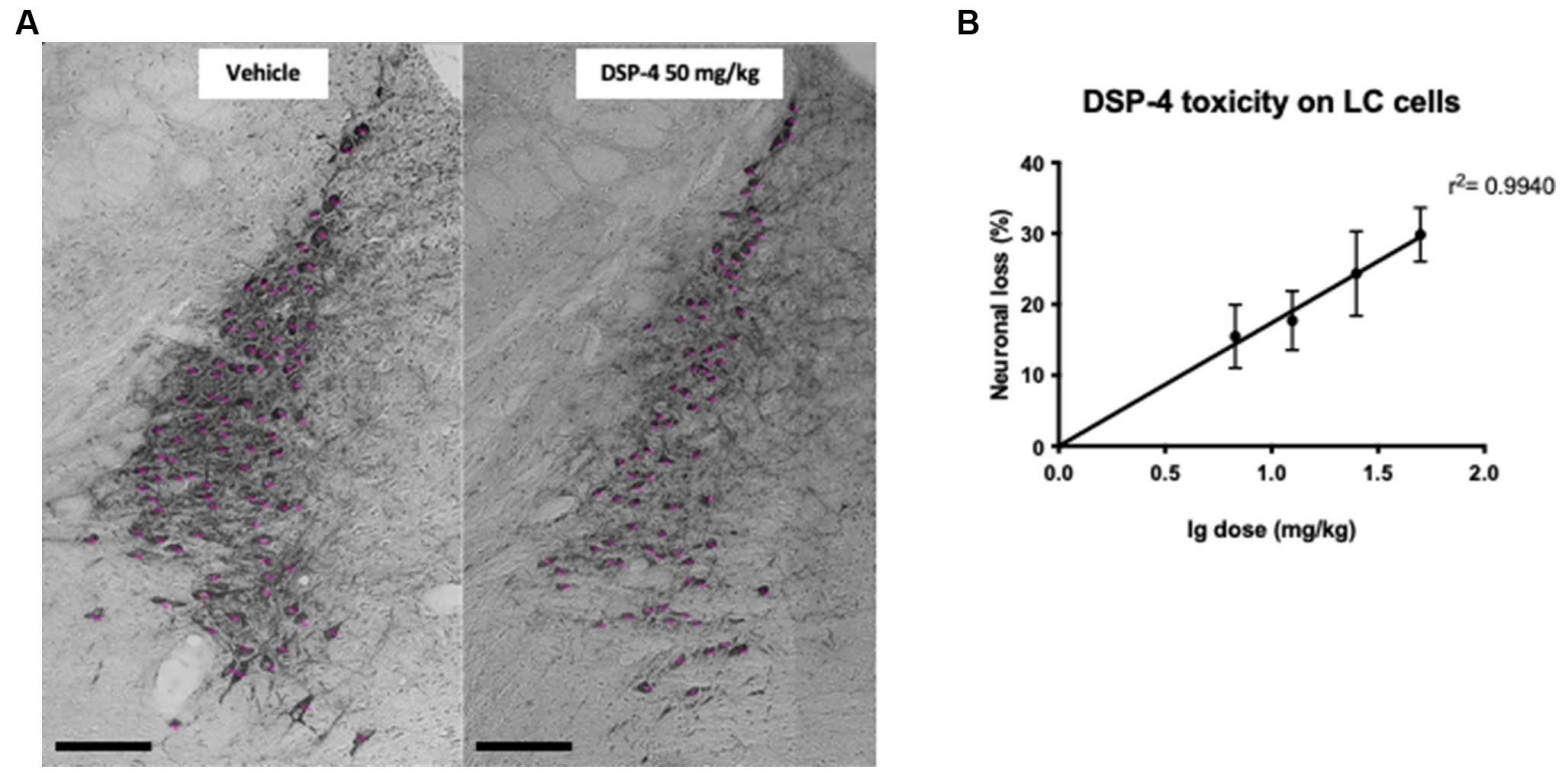
Representative photomicrographs of TH-positive cell count and DSP-4 dose–response curve. Representative photomicrographs of the tyrosine hydroxylase (TH)-positive region locus coeruleus (LC) of vehicle and DSP-4 50 mg/kg treated mice Scalebar: 100 μm. **(A)** Representation of the log linear relationship between the reduction in the number of LC neurons and the logarithm of the DSP-4 dose (*n* = 6 per dose). Shapes represent mean values, and error bars represent standard error of means (SEM) **(B)**.

Our dose-response study revealed that DSP-4 effectively reduced the number of LC noradrenergic neurons (Figure 1A). As shown in Figure 1B, we found a log-linear relationship between the dose of DSP-4 and the reduction in the number of LC neurons in the dose range used (6.25 mg/kg–50 mg/kg). Thus, we could use the simple linear regression model to determine the efficacy of the toxin (*r*^2^ = 0.9940; *p* = 0.0002) and the equation to determine the dose-response relationship: *Y* = 17.38x - 0.001460. Based on this equation, the calculated dose of DSP-4 that induced 20% cell death was 14 mg/kg.

### 3.2. DSP-4 treated animals show a lower noradrenergic innervation in prefrontal cortex

**Figure 2:**
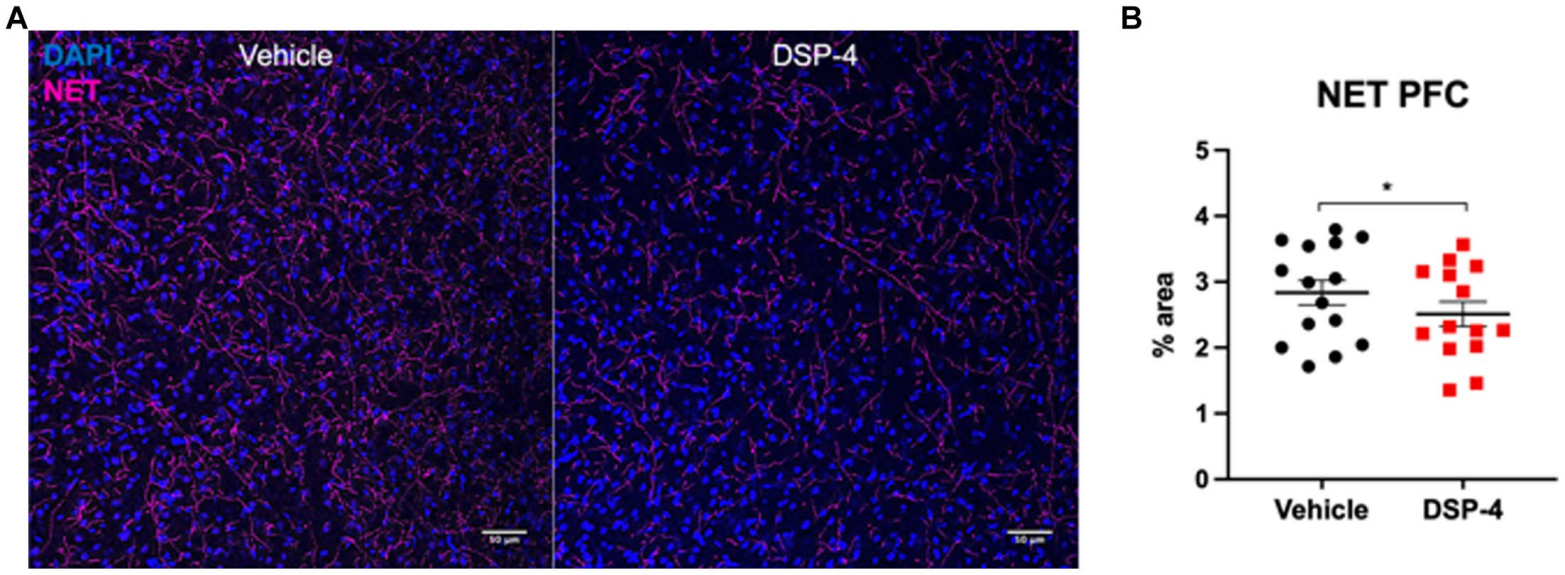
Norepinephrine transporter staining in prefrontal cortex of vehicle and DSP-4 injected animals. Representative photomicrograph of NET staining in PFC of vehicle and DSP-4 14 mg/kg injected animals **(A)**. Analysis of the area covered by NET-positive axon terminals in the PFC of vehicle and DSP-4 injected animals. Scalebar: 50 μm **(B)**. *p = 0.0410 according to Student *t*-test (*n* = 13 per treatment). Dots represent single animals; error bars represent standard error of means (SEM).

To confirm the reduction of noradrenergic signaling in our model, we performed a norepinephrine transporter (NET) staining in prefrontal cortex (PFC), brain area playing a critical role in attention and working memory (Figure 2A). As expected, 14 mg/kg DSP-4 treated animals showed a significant decrease of roughly 20% in the area covered by NET-positive axon terminals (*p* = 0.0410) (Figure 2B).

### 3.3. Age-related fear extinction and memory deficits in C57BL/6 J mice

**Figure 3:**
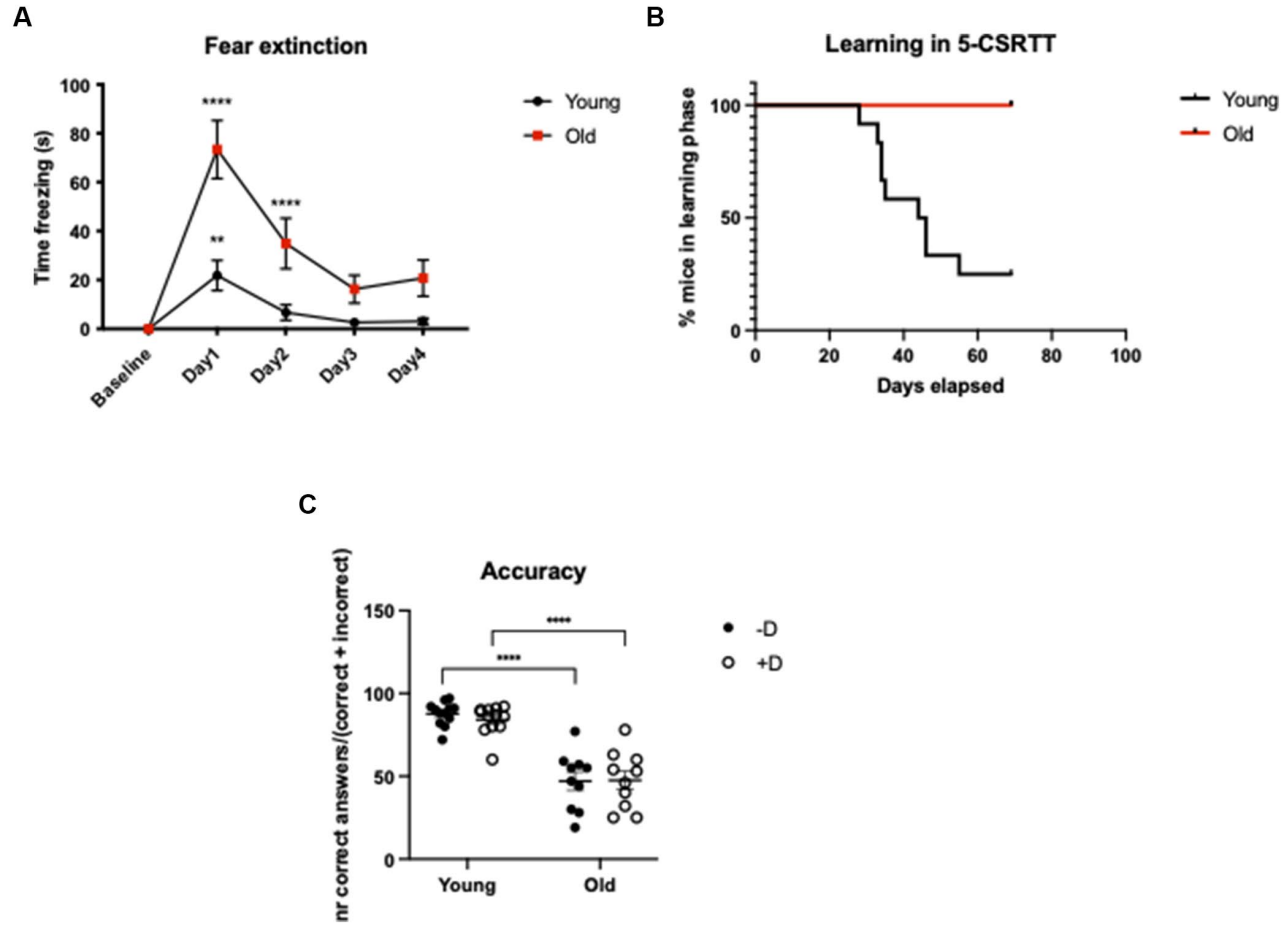
CFC and 5-CSRTT in young and old C57BL/6 J mice. CFC - Analysis of time freezing (s) in the fear extinction in young (3-months-old) and old (20-month-old) C57BL/6 J mice. ****p = <0.0001; **p = 0.0068 according to Dunnett’s multiple comparisons test (*n* = 10 per age group). Statistics refers to the comparison between the baseline and the test within the same age-group **(A)**. 5-CSRTT - Analysis of the leaning capacity **(B)** and the accuracy of young and old C57BL/6 J mice **(C)**. ****p = <0.0001 according to Šídák’s multiple comparisons test (*n* = 10–12 per age group). Presence (+D) or absence (−D) of the distractor during the test. Shapes represent mean values **(A,B)** or single animals **(C)**; error bars represent standard error of means (SEM).

In the CFC test, we did not find any difference in the baseline time spent freezing between young and old animals. After the fear conditioning protocol, both age groups showed a significant increase in freezing response (Baseline vs. Day1: young: *p* = 0.0068; old: *p* = <0.0001), which was higher in old mice. Our data from the extinction phase clearly indicate that old animals had a fear extinction deficit: while the fear memory of young animals was already extinct at day 2, old animals needed one additional day to do so (Baseline vs. Day2: young: *p* = 0.7175; old: *p* = <0.0001. Baseline vs. Day3: young: *p* = 0.9858; old: *p* = 0.0622). Overall, our data show that there is a significant difference between the two age groups in the intensity of freezing reaction and in the fear memory extinction suggesting that aging plays a key role in this phenomenon [Age: *F*_(1, 18)_ = 12.18; *p* = 0.0026. Time: *F*_(4, 72)_ = 28.87; *p* = <0.0001. Time × age: *F*_(4, 72)_ = 8.111; *p* = <0.0001] (Figure 3A; Supplementary Figure S1).

In the 5-CSRTT, we could detect a striking difference between young and old animals in terms of learning capacity. None of the old animals could reach the last phase of the test, while the 75% of the young could complete it (Log-rank (Mantel-Cox) test *p* = 0.0005) (Figure 3B). The fact that old animals were not able to reach the last phase of the test represented a complication for a complete evaluation of the distractor’s effect on attention. However, it was clear that aged animals had an impaired attention leading to reduced number of correct responses and increased number of omissions (Supplementary Figure S2). As a result, in aged animals the overall choice accuracy was lower compared to young controls (young vs. old −D, +D: *p* = <0.0001) (Figure 3C).

### 3.4. A reduced number of noradrenergic neurons contributes to memory and attention deficits

**Figure 4:**
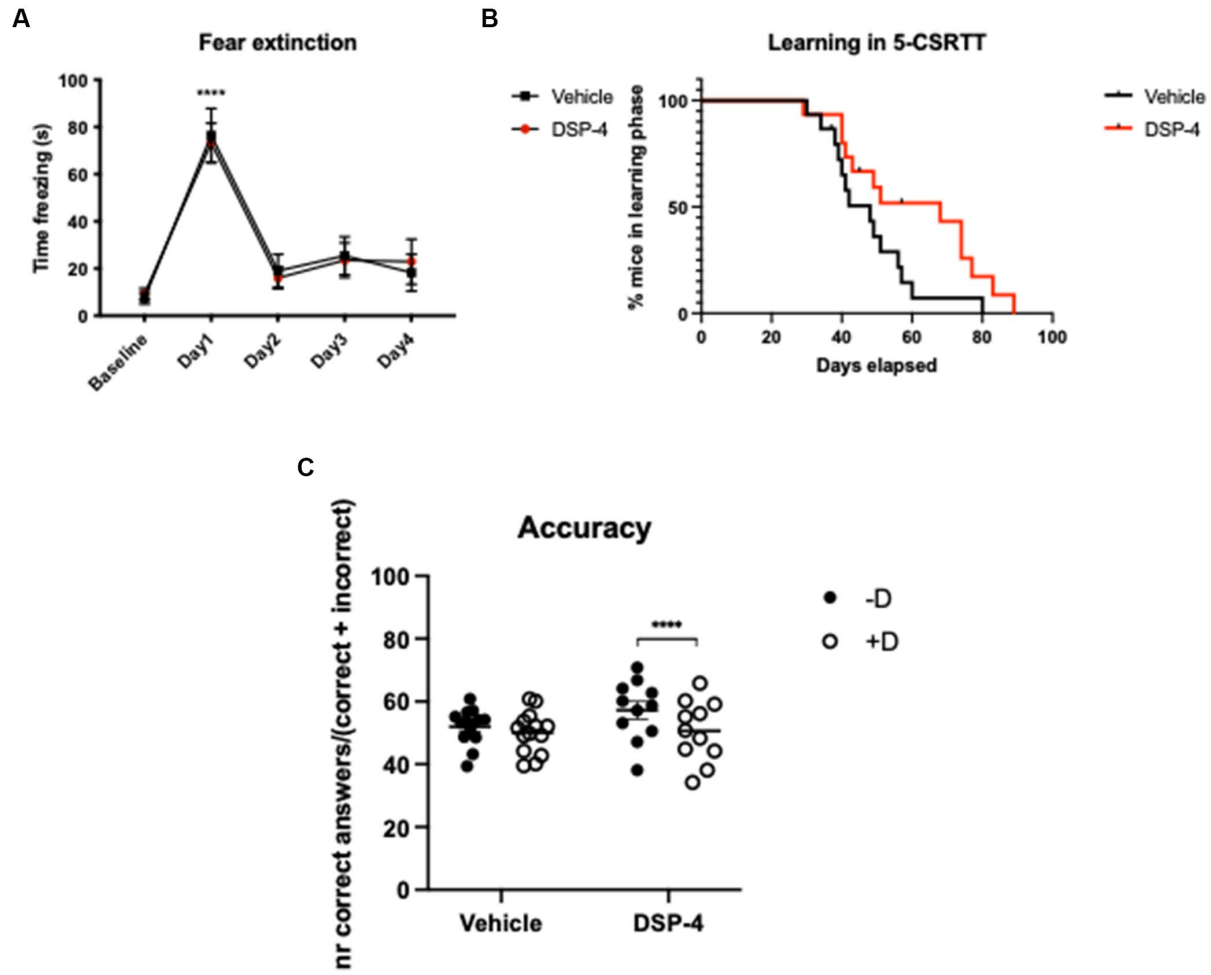
CFC and 5-CSRTT in DSP-4 and vehicle treated C57BL/6 J mice. CFC - Analysis of time freezing in the fear extinction process of DSP-4 and vehicle injected mice. ****p = <0.0001 according to Dunnett’s multiple comparisons test (*n* = 10 per treatment). Statistics refers to the comparison between the baseline and the test within the same treatment-group **(A)**. 5-CSRTT - Analysis of the leaning capacity **(B)** and accuracy **(C)** of DSP-4 and vehicle injected mice. ****p = <0.0001, according to Šídák’s multiple comparisons test (*n* = 11–15 per treatment). Presence (+D) or absence (−D) of the distractor during the test. Shapes represent mean values **(A,B)** or single animals **(C)**; error bars represent standard error of means (SEM).

In the CFC test, both DSP-4 and vehicle injected mice showed a similar increase in freezing response following the fear conditioning protocol (Baseline vs. Day1: DSP-4: *p* = <0.0001; vehicle: *p* = <0.0001) suggesting a similar fear memory formation in mice with and without LC noradrenergic neuronal depletion. Our results also indicate that fear memory extinction was not influenced by DSP-4 treatment, as we found no difference between the two groups during the course of fear extinction (Baseline vs. Day2: DSP-4: *p* = 0.8474; vehicle: *p* = 0.4811) [Treatment: *F*_(1, 18)_ = 0.00183; *p* = 0.9664. Time: *F*_(4, 72)_ = 36.62; *p* = <0.0001. Time × treatment: *F*_(4, 72)_ = 0.1546; *p* = 0.9303] (Figure 4A; Supplementary Figure S3).

In the 5-CSRTT, we could already detect a significant difference between DSP-4 and vehicle-injected animals in terms of learning capacity [Log-rank (Mantel-Cox) test; *p* = 0.0432]: DSP-4 treated animals needed more time to reach the last phase of the test compared to vehicle controls (Figure 4B). When we evaluated attention of the animals, our data demonstrated that the distractor had an effect on the number of correct and incorrect responses but not the treatment *per se*, indicating that under normal conditions the DSP-4 treated animals performed comparably with vehicle controls [Correct answers: distractor: *F*_(1, 23)_ = 31.93; *p* = <0.0001; Treatment: *F*_(1, 23)_ = 0.9214; *p* = 0.3471].

**Figure 5:**
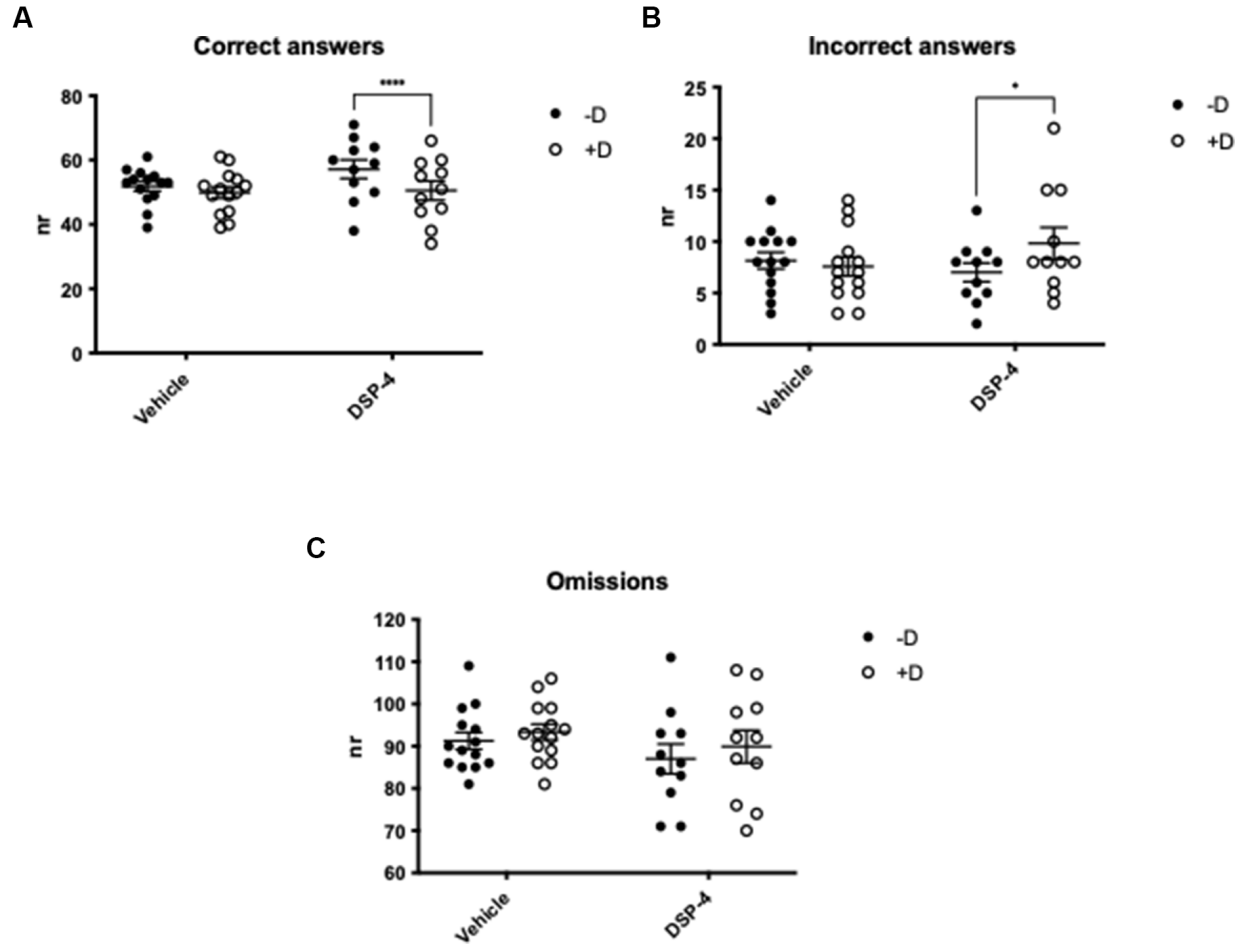
Attention in DSP-4 and vehicle treated C57BL/6 J mice. 5-CSRTT - Analysis of the number of correct **(A)**, incorrect **(B)** and omitted **(C)** answers in the presence (+D) or absence (−D) of distractor. ****p = <0.0001, *p = 0.0199 according to Šídák’s multiple comparisons test (*n* = 11–15 per treatment). Shapes represent single animals; error bars represent standard error of means (SEM).

Importantly, when testing the attention of the animals in the presence of a distractor, our data revealed that DSP-4 treated mice had a lower choice accuracy compared to vehicle controls whose performance remained stable regardless of the presence of the distractor [Treatment × distractor: *F*_(1, 23)_ = 9.648; *p* = 0.0050] (Figure 4C). The reason of the reduced choice accuracy was the increased number of incorrect responses and not an enhanced frequency of omissions [Correct answers: distractor: *F*_(1, 23)_ = 31.93; *p* = <0.0001. Treatment: *F*_(1, 23)_ = 0.9214; *p* = 0.3471. Treatment × distractor: *F*_(1, 23)_ = 9.648; *p* = 0.0050] [Incorrect answers: distractor: *F*_(1, 23)_ = 2.804; *p* = 0.1076; treatment: *F*_(1, 23)_ = 0.1743; *p* = 0.6802; treatment × distractor: *F*_(1, 23)_ = 6.382; *p* = 0.0189] [Omissions: distractor: *F*_(1, 23)_ = 4.802; *p* = 0.0388; treatment: *F*_(1, 23)_ = 1.056; *p* = 0.3147; treatment × distractor: *F*_(1, 23)_ = 0.1358; *p* = 0.7158]. The *post hoc* analysis also shown that, in the presence of the distractor, only DSP-4 treated animals gave significantly fewer correct and greater incorrect responses, while omissions were unaffected as in principle they are not influenced by the distractor (Correct answers: vehicle: *p* = 0.1306; DSP-4: *p* = <0.0001. Incorrect answers: vehicle: *p* = 0.7765; DSP-4: *p* = 0.0199. Omissions: vehicle: *p* = 0.3320; DSP-4: *p* = 0.1912) (Figures 5A–C).

### 3.5. Lack of enhanced inflammation in the LC of DSP-4 treated animals

**Figure 6:**
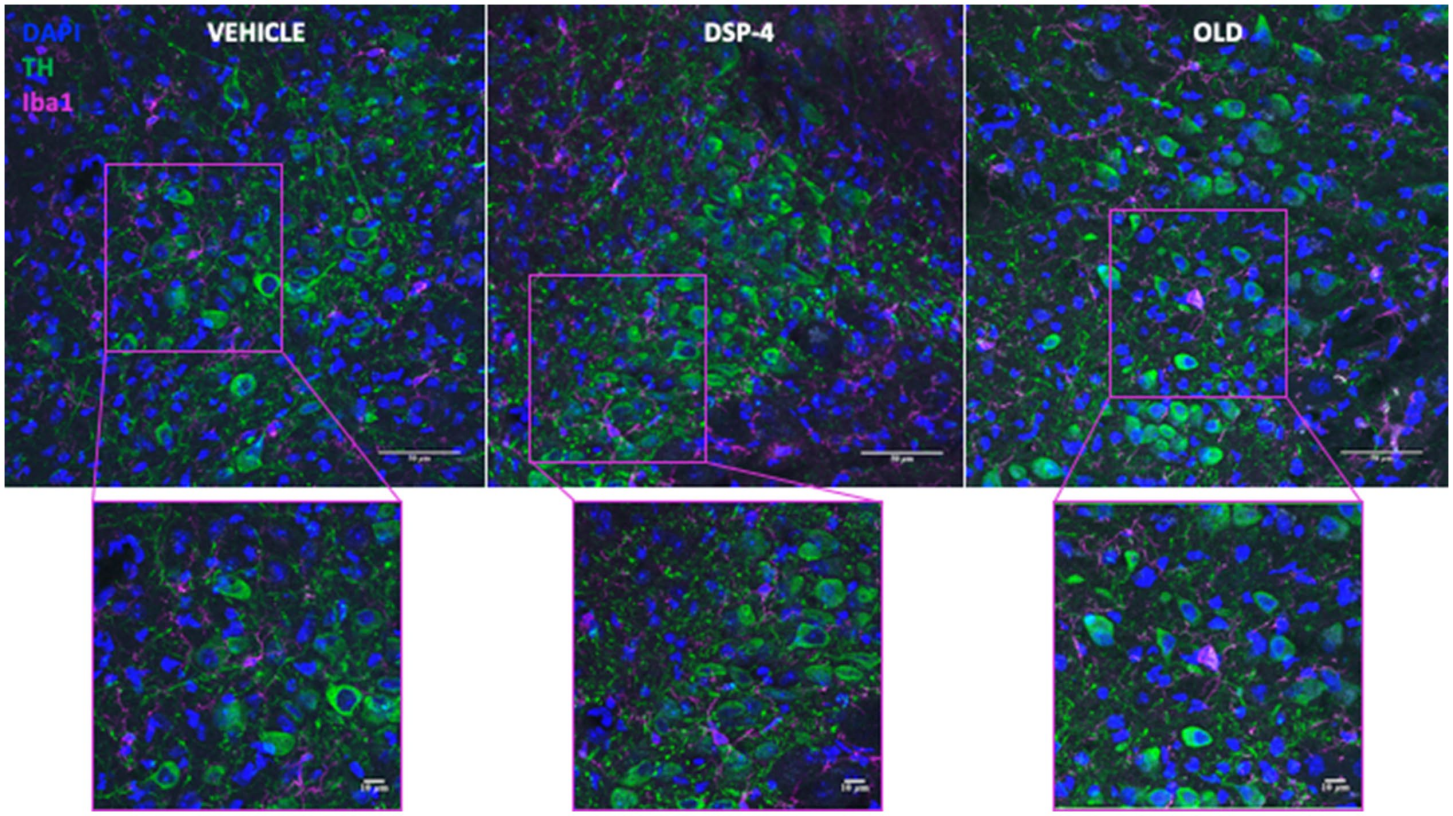
Microglia in the LC of vehicle, DSP-4 treated and old mice. Representative photomicrograph of tyrosine hydroxylase (TH) and ionized calcium-binding adapter molecule 1 (Iba1) staining in LC of a vehicle, DSP-4 injected and old animal. Scalebar: 50 μm in the whole images and 10 μm in the in the higher magnification.

Based on the recent evidence of both microgliosis and increased inflammatory proteins in the LC of old animals (Evans et al., 2021), we performed an immunohistochemical staining for the microglial marker ionized calcium-binding adapter molecule 1 (Iba1), and the tumor necrosis factor alpha (TNFα) (Figure 6; Supplementary Figure S4).

**Figure 7:**
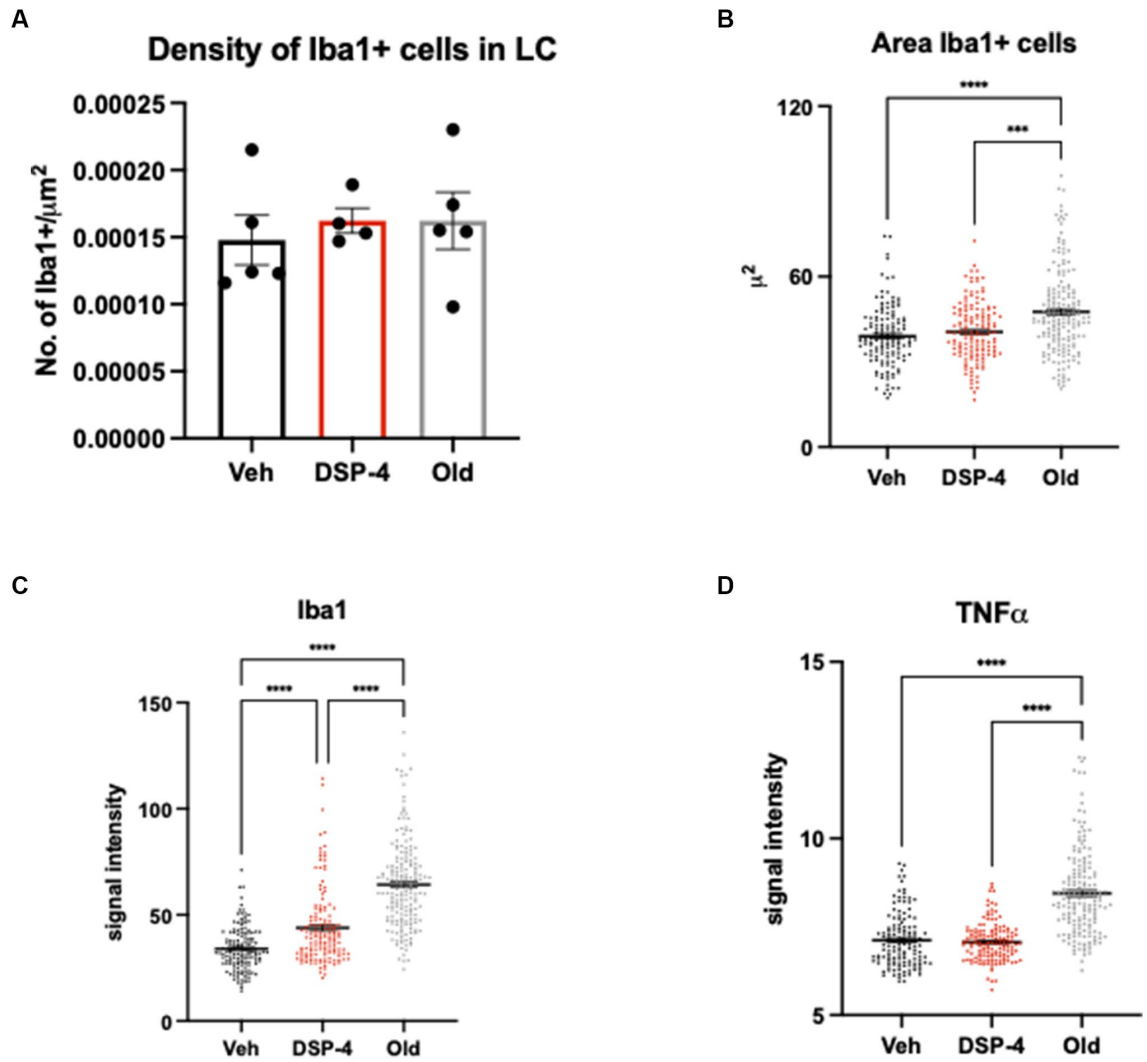
Inflammatory profile in the LC of vehicle, DSP-4 treated young vs. old animals. Density of Iba1-positive microglia within the LC of vehicle, DSP-4 treated young and old animals (*n* = 4–5 per genotype) **(A)**. Analysis of Iba1-positive microglia cell body area. ***p = 0.0002; ****p = <0.0001 according to Dunn’s multiple comparisons test **(B)**. Analysis of Iba1 **(C)** and TNFα **(D)** signal intensity in microglia cells. ****p = <0.0001 according to Dunn’s multiple comparisons test (*n* = 135–181 per group). Dots represent single animals **(A)** or individual cells **(B–D)**; error bar represents standard error of means (SEM) **(A)**, lines represent median **(B–D)**.

Our data did not show an increased microglia density neither in DSP-4 treated nor in old animals compared to young vehicle controls [*F*_(2, 11)_ = 0.2176; *p* = 0.8078. Veh vs. DSP-4: *p* = 0.8488. Veh vs. Old: *p* = 0.8329. DSP-4 vs. Old: *p* = >0.9999] (Figure 7A). We could detect an increased area of Iba1-positive microglia cells in old animals compared to both vehicle and DSP-4 treated animals; however no difference was found between vehicle and DSP-4 treated animals (*p* = <0.0001. Veh vs. DSP-4: *p* = 0.6141. Veh vs. Old: *p* = <0.0001. DSP-4 vs. Old: *p* = 0.0002) (Figure 7B). Interestingly, we could detect a progressive increase in the Iba1 signal intensity dependent on the treatment and the age (*p* = <0.0001. Veh vs. DSP-4: *p* = <0.0001. Veh vs. Old: *p* = <0.0001. DSP-4 vs. Old: *p* = <0.0001) (Figure 7C). Contrarily, the TNFα signal intensity was affected only by age, with a clear increase in old compared to vehicle or DSP-4 treated animals (*p* = <0.0001. Veh vs. DSP-4: *p* = > 0.9999. Veh vs. Old: *p* = <0.0001. DSP-4 vs. Old: *p* = <0.0001) (Figure 7D).

## 4. Discussion

Although detailed stereological analyses of the aging brain revealed that the histological structure of the brain is largely preserved, there is a significant neuronal loss in several brain areas such as the (LC) (Flood and Coleman, 1988). In patients with neurodegenerative diseases such as Alzheimer’s disease (AD) (German et al., 1992) or with mild cognitive impairment (Grudzien et al., 2007) a substantial loss of LC neurons has been clearly demonstrated. There is a significant loss of LC neurons even in the prodromal symptom-free phase of AD (Arendt et al., 2015), with further reductions in the successive Braak stages (Theofilas et al., 2017). Reduced noradrenergic signaling is known to contribute to the pathogenesis of Alzheimer’s disease. The reduction in the number of LC noradrenergic neurons is more moderate in normal aging. It was not known whether this approximately 20% loss of LC neurons had any effect on cognitive function. In our present work, we now show that a reduction in LC noradrenergic neurons in young animals, i.e., in the absence of all other age-related histological changes such as neuroinflammation, impairs cognitive functions.

Aging is known to be associated with a progressive decline in episodic memory (Levine et al., 2002), working memory (Gazzaley et al., 2005), speed of information processing and task switching. An age-related decline in attention could significantly contribute to these deficits (Robertson, 2013). Some studies have also proven how aging selectively impairs the process of memory extinction and the capacity to use context to modulate learned responses to threat (Battaglia et al., 2018). These cognitive domains that seem to be deeply influenced by aging have also been shown to be heavily regulated by the noradrenergic system (Arnsten et al., 1997; Seo et al., 2021). For these reasons we selected a battery of models (CFC and 5-CSRTT) sensitive to both age-related decline and noradrenergic signaling. In this way, we could selectively investigate memory formation and extinction, working memory and attention in our animals.

Our behavioral data pointed out that old C57BL/6 J mice experience difficulty extinguishing fearful memory compared to young controls in the contextual fear conditioning test (Figure 3A; Supplementary Figure S1). Different studies confirmed our data and provided us with additional insights to explain what indeed happens during aging (Shoji et al., 2016; Battaglia et al., 2018; Yanai and Endo, 2021). For example, Shoji et al. (2016) conducted a large-scale analysis of age effects on behavior in C57BL/6 J wild-type mice. In particular, they examined the mice in the startle response/prepulse inhibition test and in the fear conditioning test, both known to be associated with the activity of the noradrenergic system (Kokkinidis and Anisman, 1978; Saitoh et al., 1986; Fendt et al., 1994; Giustino and Maren, 2018), finding an age-related increased prepulse inhibition and especially an increase in freezing behavior. Therefore, they suggested that this could be explained by an age-related increase in generalized fear/anxiety or age-related deficits in the ability to discriminate between contexts. Moreover, a study from Yanai and Endo (2021) also showed results consistent with our own; he also hypotheses that, based on their data, the intense freezing observed in older aged mice may reflect elevated anxiety states and/or reduced locomotor activity.

Importantly, in our study we could also demonstrate that old C57BL/6 J animals show a significant impairment in working memory in the 5-CSRTT compared to young controls, which was associated with lower choice accuracy (Figures 3B,C). It is well known how noradrenaline modulates the balance of cortical excitation and inhibition and how these neuromodulatory adjustments facilitate the selective processing of prioritized information. Thus, by disrupting the coordination in attention networks, age-related LC degeneration can impair the neural processing (Dahl et al., 2022). Contextually, a study in humans from Lee et al. (2018) showed how arousal, which in young adults increased processing of salient stimuli and decreased processing of non-salient stimuli, in older adults increased processing of both low and high-salience stimuli. Older adults also showed a drop in LC functional connectivity. Thus, the lower choice accuracy that we associated with aging could be due to an impaired arousal and an age-related altered processing of non-salient stimuli (Lee et al., 2018). Additionally, a recent human study indicated that cognitive components contributing to distractibility follow different trajectories with aging and that increased distractibility in older adults seems to result from a dominance of involuntary attention processes (Hoyer et al., 2022).

The cause of these age-related pathologies is not fully understood, although this is the key to successful therapy or prevention of dementia and neurodegenerative disorders.

To clarify whether and to what extent a specific element of brain aging—in our study a reduction in noradrenergic signaling due to the loss of LC noradrenergic neurons—is responsible for these cognitive deficits, we depleted LC neuronal numbers by treating young animals with 14 mg/kg DSP-4 toxin and tested them 60 days after the treatment. This model allowed us to selectively test the effect of a similar loss of neurons as found in the aged brain, but in the absence of other age-related pathologies such as neuroinflammation.

Consistently with the literature (Evans et al., 2021), old mice showed an increased pro-inflammatory profile in the LC characterized by increased microglial volume and enhanced Iba1 and TNFα levels. On the other hand, DSP-4 treated mice showed only a moderate increase in Iba1 levels, while in other parameters they were similar as vehicle controls (Figure 7). We could therefore exclude that an extensive inflammatory change due to the neuronal death was responsible for the observed memory and attention deficits. The fact that we did not find enhanced pro-inflammatory glial activity is probably due to the relatively low dose of DSP-4. Previous studies, typically using 50 mg/kg of DSP-4, reported enhanced neuroinflammation and oxidative stress in the LC and in its main target areas (Iannitelli et al., 2023).

It is important to note, that although the extent of neuronal loss in our model and during aging is similar, the underlying mechanisms are substantially different. DSP-4 is able to cross the blood–brain barrier and block noradrenaline release at the nerve terminals by causing an initial inhibition of the noradrenaline transporter (NET) (Dudley et al., 1990; Ross and Stenfors, 2015). Administration of 50 mg/kg DSP4 leads to a rapid depletion of noradrenaline levels, followed by a degeneration of noradrenergic axon terminals within 24 h (Grzanna et al., 1989). The number of noradrenergic cell bodies remains stable for at least 18 days after the treatment, although these cells show expression changes indicative of neurodegeneration and reduced cell identity (Iannitelli et al., 2023). A reduction also in the number of LC noradrenergic neurons is present first 23 days after the DSP-4 treatment (Li et al., 2018) Although the reduction in neuronal numbers persists for at least 1-year, almost complete regeneration of the axons was observed 6 months after the treatment (Fritschy et al., 1992). On the other hand, there are several factors, which contribute to the increased vulnerability of noradrenergic neurons to neurodegenerative changes in aging: dopamine toxicity due to increased oxidative stress during the noradrenaline synthesis, high neuronal iron content, autonomous pacemaker activity (Sanchez-Padilla et al., 2014), very high axonal arborization size (Morrison et al., 1979), which together lead to energetic failure (Giguère et al., 2018), are all thought to contribute to the slow decline of LC neurons. Nevertheless, both aging and treatment of young mice with 14 mg/kg DSP-4 resulted in a similar decline in noradrenergic signaling.

As expected, our model revealed a significant decrease in the density of noradrenergic axon terminals in the PFC (Figure 2). We selected this brain region because the PFC is crucial in mediating the working memory and attentional functions, for which the key importance of noradrenaline has been extensively demonstrated (Aston-Jones et al., 1991, 1994; Arnsten et al., 1997; Sara, 2009). PFC receives noradrenergic projections selectively from the LC. The noradrenergic system in the brain is composed of two primary ascending projections that originate from the brainstem: the dorsal noradrenergic bundle (DNB) and the ventral noradrenergic bundle (VNB). The DNB originates from A6 LC, and it functions as the principal site of norepinephrine production in the central nervous system. It sends projections to the cerebral cortex, hippocampus, and cerebellum exclusively, while the VNB, originating from nuclei in the pons and medulla, sends projections to innervate mostly amygdala, hypothalamus, and areas of the midbrain and medulla (Weinshenker and Schroeder, 2007; Montoya et al., 2016; Rho et al., 2018). Thus, the reason for the lower density of noradrenergic terminals in the PFC of DSP-4 treated mice must be the reduced number of LC noradrenergic neurons. Indeed, in our previous study, we demonstrated that the neuronal loss in the LC during aging similarly affects the density of axon terminals in all the efferent regions analyzed [cortex, hippocampus, amygdala, and hypothalamus (Gargano et al., 2021)]. We also excluded the involvement of the peripheral sympathetic system in the observed cognitive deficits, since NE (and in general catecholamines) are unable to cross the blood-brain-barrier. In fact, the blood-brain-barrier is both a physical and an enzymatic barrier, containing the catecholamine metabolizing enzymes monoamine oxidase and catechol-O-methyltransferase (Bertler et al., 1963; Hamberger and Masuoka, 1965; Bertler et al., 1966; Bartholini et al., 1971). Although the blood-brain-barrier is fairly permeable to NE at birth (Glowinski et al., 1964), its permeability begins to decrease as early as 2 weeks after birth (Loizou, 1970), and becomes fully competent in adulthood (Axelrod et al., 1959; MacKenzie et al., 1976; Kostrzewa, 2007).

Our data did not show any significant difference between DSP-4 treated and vehicle control animals in either the process of fear learning or extinction, indicating that the age-related neurodegeneration of LC neurons alone is not liable for the deficit in fear extinction characteristic of old age (Figure 4A). This could be explained by the major role played by other brain regions in contextual conditioning, for instance the hippocampal dentate gyrus. Indeed, despite the β-adrenergic-mediated modulation of hilar interneurons is necessary to promote aversive generalization (Seo et al., 2021), a reduction as small as that associated with aging is probably not sufficient to disrupt the whole process. In contrast to our mice treated with 14 mg/kg DSP-4, mice repeatedly treated with 50 mg/kg DSP-4 showed deficits in contextual fear memory (Chalermpalanupap et al., 2018). Not surprisingly, the reduction in the NET-positive noradrenergic axon terminals was much higher using higher amount of DSP-4: it was 50% in Chalermpalanupap’s study and 20% in our study. We hypothesize that a moderate reduction in noradrenergic signaling, which occurs during normal, healthy aging, does not significantly affect memory abilities. A more severe reduction, such as that observed in Alzheimer’s and Parkinson’s disease, may impair learning and memory *per se*.

Yet, when comparing DSP-4 treated and vehicle animals in the 5-CSRTT, we found that DSP-4 treated animals needed significantly more time to learn the task and to reach the last phase of the test compared to vehicle injected controls, indicating an impaired working memory (Figure 4B). Interestingly, our data also show that, when a distractor was introduced in the test, the DSP-4 treated mice exhibited a poorer choice accuracy compared to controls which did not seem to be disturbed by it (Figures 4C, 5). Our data also showed that, in both vehicle and DSP-4 treated groups, the presence of the distractor influenced the number of correct/incorrect answers but not the number of omissions. This could be explained by the fact that the distractor does not impact the drive of the animal *per se* (which is mostly due to the food restriction) but influences the choice accuracy during test. It is also important to remember that DSP-4 treated animals are considered young and that the only brain region altered the way aging would is the LC. Therefore, these data seem to be particularly interesting as, while DSP-4 treated animals did not show a major impairment as old animals did, their performance was compromised compared to vehicle controls. Thus, these data demonstrated that even a small reduction in noradrenergic signaling is able to impact working memory and attention as these cognitive domains are majorly controlled by the noradrenergic system.

In conclusion, we have found that a reduction in LC neurons may directly contribute to impaired working memory and greater distractibility in the performance of attentional tasks. Thus, even in the absence of other age-related changes in the brain, a reduced number of LC noradrenergic neurons *per se* impairs cognitive functions. Taken together, our findings help to elucidate the role of reduced noradrenergic signaling in age-related cognitive deficits and provide a druggable target for alleviating negative symptoms of brain aging.

## Data availability statement

The raw data supporting the conclusions of this article will be made available by the authors, without undue reservation.

## Ethics statement

The animal study was approved by Landesamt fuer Natur, Umwelt und Verbraucherschutz Nordrhein-Westfalen. The study was conducted in accordance with the local legislation and institutional requirements.

## Author contributions

AG: Formal Analysis, Investigation, Methodology, Writing – original draft, Writing – review & editing. BO: Methodology, Writing – original draft. MP: Investigation, Methodology, Writing – original draft. AZ: Funding acquisition, Writing – review & editing. AB-G: Conceptualization, Funding acquisition, Resources, Supervision, Writing – original draft, Writing – review & editing.

## Funding

The author(s) declare financial support was received for the research, authorship, and/or publication of this article.

The work was funded by the Deutsche Forschungsgemeinschaft (DFG, German Research Foundation)—project numbers 426320013 and 461228630 to AB-G and to AZ under project number 324087152 as well as under Germany’s Excellence Strategy—EXC2151—390873048.

## Conflict of interest

The authors declare that the research was conducted in the absence of any commercial or financial relationships that could be construed as a potential conflict of interest. The author(s) declared that they were an editorial board member of Frontiers, at the time of submission. This had no impact on the peer review process and the final decision.

## Publisher’s note

All claims expressed in this article are solely those of the authors and do not necessarily represent those of their affiliated organizations, or those of the publisher, the editors and the reviewers. Any product that may be evaluated in this article, or claim that may be made by its manufacturer, is not guaranteed or endorsed by the publisher.

## Abbreviations

5-CSRTT: 5-choice serial reaction time test
AD: Alzheimer’s disease
BW: Body weight
CFC: Contextual fear conditioning
DAB: Diaminobenzidine
DSP-4: N-(2-Chloroethyl)-N-ethyl-2-bromobenzylamine hydrochloride
FI: Food intake
Iba1: Ionized calcium-binding adaptor molecule 1
LC: Locus coeruleus
NET: Noradrenaline transporter
PD: Parkinson’s disease
PFC: Prefrontal cortex
TH: Tyrosine hydroxylase
TNFα: Tumor necrosis factor alpha
WT: Wild-type
