# Supplementary material for: Possible role of locus coeruleus neuronal loss in age-related memory and attention deficits
Source: Front Neurosci. 2023 Aug 24;17:1264253. doi: 10.3389/fnins.2023.1264253 (PMC10492095; doi:10.3389/fnins.2023.1264253)

Supplementary Material

# Supplementary Figures

**Supplementary Figure 1.** **Fear extinction in young and old C57BL/6J mice.**

Analysis of time freezing (s) during the first **(A)**, second **(B)**, third **(C)** and last **(D)** day of fear extinction process of young (3-months-old) and old (20-month-old) C57BL/6J mice. Shapes represent mean values; error bars represent standard error of means (SEM).

**Supplementary Figure 2.** **Attention in young (3-months-old) and old (20-month-old) C57BL/6J mice.**

5-CSRTT - Analysis of the number of correct **(A)**, incorrect **(B)** and omitted **(C)** answers in the presence (+D) or absence (-D) of distractor. * *p* = 0.0298 **(A)** and 0.0407 **(C)** according to Šídák's multiple comparisons test (n = 10 – 12 per age group). Shapes represent single animals; error bars represent standard error of means (SEM).

**Supplementary Figure 3.** **Fear extinction in DSP-4 and vehicle treated C57BL/6J mice.**

Analysis of time freezing (s) during the first **(A)**, second **(B)**, third **(C)** and last **(D)** day of fear extinction process of DSP-4 and vehicle treated C57BL/6J mice. Shapes represent mean values; error bars represent standard error of means (SEM).

**Supplementary figure 4: Iba1 and TNFα staining in the LC of vehicle, DSP-4 treated and old mice.**

Representative photomicrograph of tyrosine hydroxylase (TH), ionized calcium-binding adapter molecule 1 (Iba1) and tumor necrosis factor alpha (TNFα) staining in LC of a vehicle, DSP-4 injected and old animal. Negative controls (NC) were stained only with the secondary antibodies. Scalebar: 50 μm.


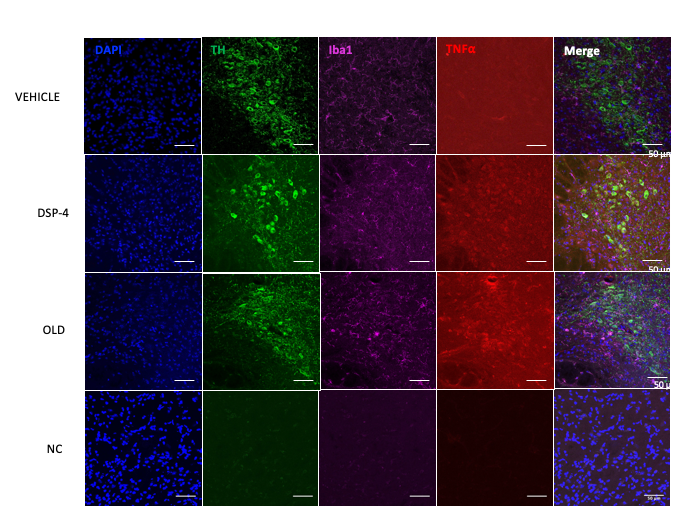

Supplement: Supplementary file 1 [file Data_Sheet_1.docx]
